# Pharmacological potential of micheliolide: A focus on anti-inflammatory and anticancer activities

**DOI:** 10.1016/j.heliyon.2024.e27299

**Published:** 2024-03-05

**Authors:** Jalal Uddin, Mehwish Fatima, Ammara Riaz, Ghulam Mustafa Kamal, Abdullatif Bin Muhsinah, Abdul Razzaq Ahmed, Ramsha Iftikhar

**Affiliations:** a Department of Pharmaceutical Chemistry, College of Pharmacy, King Khalid University, Asir, 61421, Saudi Arabia; b Department of Zoology, Faculty of Life Sciences, Government College University, Faisalabad, 38000, Pakistan; c Department of Life Sciences, Khwaja Fareed University of Engineering and Information Technology, Rahim Yar Khan, 64200, Pakistan; d Institute of Chemistry, Khwaja Fareed University of Engineering & Information Technology, Rahim Yar Khan, 64200, Pakistan; e Department of Pharmacognosy, College of Pharmacy, King Khalid University, Asir, 61421, Saudi Arabia; f Department of Prosthodontics, College of Dentistry, King Khalid University, Abha, 61421, Saudi Arabia; g School of Chemistry, University of New South Wales, 2033, Sydney, Australia

**Keywords:** Micheliolide, A sesquiterpene lactone, *Magnolia grandiflora* L, Anticancer, Anti-inflammatory

## Abstract

Micheliolide (MCL) is a chief constituent of plants such as *Magnolia grandiflora* L.*, Michelia compressa* (Maxim.) Sarg*.* and *Michelia champaca* L. It is known to exhibit significant anticancer activity by various scientific investigations. This review aims to emphasize the anticancer and antiinflammatory activities of MCL. In this review, we summarized the published data in peer-reviewed manuscripts published in English. Our search was based on the following scientific search engines and databases: Scopus, Google Scholar, ScienceDirect, Springer, PubMed, and SciFinder, MCL possesses a broad spectrum of medicinal properties like other sesquiterpene lactones. The anticancer activity of this compound may be attributed to the modulation of several signaling cascades (PI3K/Akt and NF-κB pathways). It also induces apoptosis by arresting the cell cycle at the G1/G0 phase, S phase, and G2/M phase in many cancer cell lines. Very little data is available on its modulatory action on other signaling cascades like MAPK, STAT3, Wnt, TGFβ, Notch, EGFR, etc. This compound can be potentiated as a novel anticancer drug after thorough investigations *in vitro*, *in vivo*, and *in silico*-based studies.

## Graphical abstract

Graphical abstract depicting the biological resources, chemical structure, and mechanism of action of Micheliolide.

**Figure:**
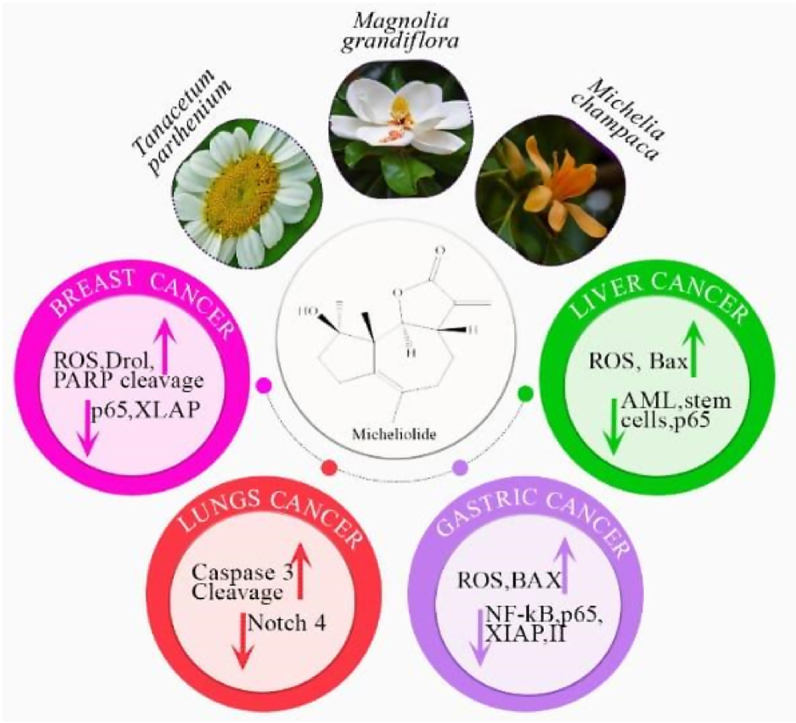

## 1 Introduction

Nature has always endeavored mankind with the extensive and infinite treasure of molecular entities, which can be utilized enormously to develop newer drug candidates, pharmacophores, and chemical scaffolds to develop novel drugs for treating many diseases with different origins [1,2]. The chemical entities extracted from natural resources can be termed “Natural Products” (NPs) and are referred to as the end products of gene expression, *i.e.,* secondary metabolites from natural sources [3,4]. NPs are the most persistently successful sources of drug innovation and have been the backbone of conventional remedies worldwide [2]. They have also been an intrinsic part of culture and history [1,5]. NPs from minerals, microbes, plants, and animals have long been anticipated as remedies against diseases for human beings [4]. NPs from plants have long been valuable sources of medicinal components, and up till now, numerous pharmacological products have plant-based origins [6]. Screenings of therapeutic plants have led to the discovery of potent sources of antioxidants [7], anti-cancer [[8], [9], [10], [11], [12]], anti-inflammatory [13], and neuroprotective mediators [14]. A vast family of plant-based compounds, “Sesquiterpene Lactones (SLs),” shows significant pharmacological properties, including the eradication of cancer as well as controlling inflammation [15,16]. Guaianolides is a subdivision of SLs with a tricyclic 5,7,5-ring system [17].

**Fig. 1:**
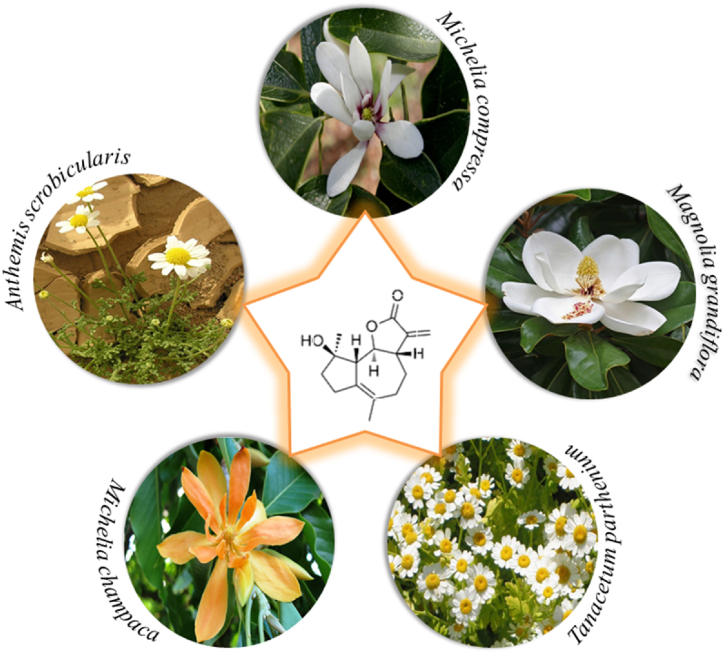
Natural sources of Micheliolide.

MCL is a potentially stable sesquiterpene lactone [18], which can be isolated from *Magnolia grandiflora* L*., Michelia champaca* L*.,* and *Michelia compressa* (Maxim.) Sarg*.* MCL and its Michael adduct dimethylaminomicheliolide (DMAMCL) display significant cytotoxicity against tumors [[18], [19], [20], [21], [22], [23], [24]]. MCL is known to be firstly isolated by Ogura, Cordell [25] from *Michelia compressa* (Maxim.) Sarg. as a cytotoxic active component*.* MCL is also a main biologically active constituent of *Michelia champaca* L*.* [23,[26], [27], [28], [29], [30], [31], [32], [33]], *Magnolia grandiflora* L*.* [20], *Tanacetum parthenium* Sch. Bip. [34], *Anthemis scrobicularis* Yavin [35], and *Michelia compressa* (Maxim.) Sarg [8,23,[25], [26], [27], [28], [29], [30], [31]]. as shown in Fig. 1. *Magnolia or Michelia* is a genus with 90 species belonging to the family Magnoliaceae [20]. Chinese traditional medicines also use an alternative cancer treatment [36,37]. Indigenous people in China use Michelia species to cure certain cancers [27], *e.g.,* *Magnolia champaca* var. champaca has been utilized to cure leukemia and *Aldama hypoleuca* (S.F.Blake) E.E.Schill. & Panero is used for the treatment of carcinomatous sores.

Moreover, *Michelia champaca* L*.* has been used for curing abdominal lumps in India [38]. *Magnolia kobus* DC*., Michelia compressa* (Maxim.) Sarg and *Magnolia grandiflora* L have shown anticancer activity against several tumor systems in several scientific studies [39]. Table 1 shows some common sources of MCL along with their pharmacological efficacy in the case of different diseases. The plant-derived sesquiterpene lactone MCL was recently found to possess promising antileukemic activity, including the ability to target and kill leukemia stem cells.

**Table 1:** Biological sources of Micheliolide and their efficacy in case of several diseases.

Botanical name	English name	Parts used	Extraction solvent	Disease/Function	References
*Magnolia grandiflora*L	Southern Magnolia or bull bay	Leaves	Acetone	Used to treat headache, hypertension, fever, diarrhea, and rheumatism. Anticancer	[20]
*Michelia compressa* (Maxim.) Sarg.		Root bark	Chloroform fraction of methanol extract	Anticancer, antineoplastic	[8,23,[25], [26], [27], [28], [29], [30], [31]]
*Michelia Champaca* L.	Golden Champa	Root wood, Stem bark	Ethanol,	Anticancer, anti-inflammatory, antifungal, antipyretic, antioxidant, antibacterial, anti-thrombotic, Hepatoprotective, anti-hyperglycemic, anti-fertility, dysmenorrhea, fever, ulcers, wounds, and skin diseases	[23,[26], [27], [28], [29], [30], [31], [32], [33]]
*Anthemis cretica* subsp. *cretica L.*	–	Aerial parts	Methanol	Antifungal, schistosomicidal, antioxidant, antiplasmodial, antitumor, anthelminthic, cytotoxic, analgesic activities, phytotoxic	[35]
*Tanacetum parthenium* Sch.Bip.	Feverfew	–	–	Antimicrobial, anti-inflammatory, antiviral, antibacterial, and antifungal	[34]

**Fig. 2:**
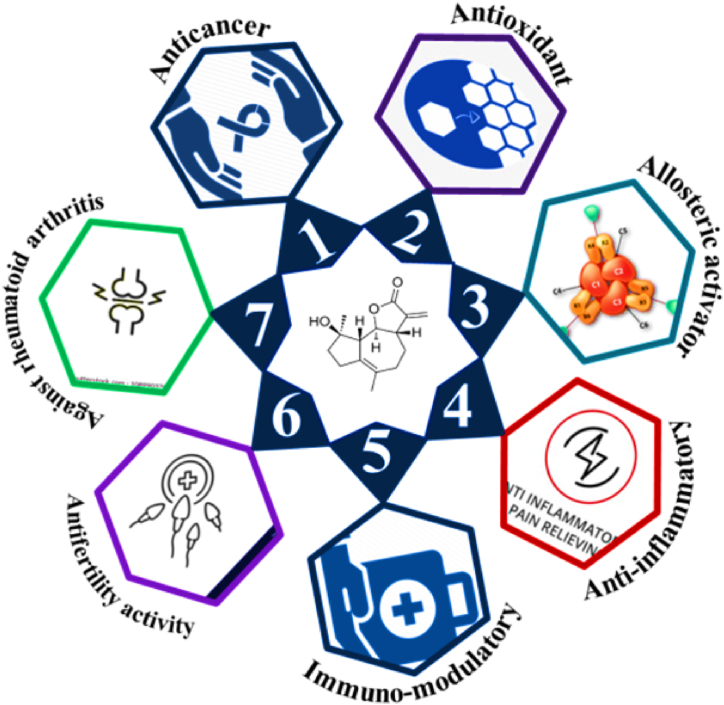
Biological activities of the Micheliolide.

Nature-derived biologically active guaniolide sesquiterpene lactone, MCL, has exhibited numerous therapeutic properties such as anticancer, antioxidant, allosteric activation, anti-inflammatory, immune-modulatory, anticancer, antifertility activities, and rheumatoid arthritis. (Fig. 2).

Until now, no one has summed up MCL's anticancer and antiinflammatory activities in the form of a review article. Thus, this review aims to summarize MCL's anticancer and antiinflammatory activities.

## 2 Anti-inflammatory activity of MCL

Inflammation is a complicated system arising in retort to tissue injury or damage caused by some infection. This process is associated with the onset of different pathological states like cancer development, cardiovascular ailments, and autoimmune diseases [40,41]. Sepsis is a multifaceted metabolic and physiological reaction to infection followed by multiple organ failure leading to death [42]. Despite the better result of sepsis due to more and timely usage of glucocorticosteroids, antibiotics, and compassionate care, the prevalence of severe sepsis is still on the rise [43,44]. Primary sepsis-causing microorganisms are gram-negative (G-) bacteria. The main part of G-bacteria's cell wall, lipopolysaccharide, is a ligand of Toll-like receptor 4 (TLR4); and provokes an unrestrained inflammatory response, even leading to death [45]. Currently, there is no clinically available auspicious therapeutic substance [46]. Although lipopolyamines [47] and synthetic anti-lipopolysaccharide peptides (SALPs) [48,49] exhibit tremendous neutralizing character against LPS, there is also an urgent need for immunomodulatory substances to resist the devastating inflammatory response in septic patients. Harmful side effects associated with non-steroidal anti-inflammatory drugs (NSAIDs) provoke an urge to discover harmless substitutes [50]. MCL has been described to show anti-inflammatory activity and reduces LPS-stimulated synthesis of interferon β (IFN-β), tumor necrosis factor-alpha (TNF-α), monocyte chemotactic protein 1 (MCP-1), Interleukin 6 (IL-6), Interleukin 10 (IL-10), and IFN-β in dendritic cells, Monocyte/macrophage cell line (RAW 264.7), human monocytic cell, human CD14^+^ monocytes THP-1 (leukemia cell line) and primary peritoneal macrophages [51].

MCL has been found to mitigate PI3K/Akt/p70S6K and Nuclear factor-kappa B (NF-κB) stimulation, specially obstructing the addition of phosphate to p70S6K (Thr389), Akt (Ser473), and IκBα (Ser32/36) [26]. These results give a different insight into regulating LPS-induced inflammatory reactions indicating an auspicious anti-septic character of MCL in the severe peritonitis mouse model. Hence, to cure lethal LPS-induced septic shock, MCL is a promising drug aspirant to develop potent anti-inflammatory and immunosuppressive substances.

MCL mitigated inflammatory responses and accumulation of lipids in LM (lipid molecule)-influenced L02 and AML12 cells via up-regulating Peroxisome proliferator-activated receptor gamma (PPAR-γ) and reducing p–NF–κB/p65 and Anti-phosphorylated-IκBα (*p*-IкBα), thus decreasing lipotoxicity by obstructing the NF-κB pathway. MCL improves hepatic steatosis via upregulation of PPAR-γ expression, thus deterring NF-κB-induced inflammation and triggering AMPK/mTOR-reliant autophagy [52]. MCL distinctly promotes HO-1 (heme oxygenase-1, an antioxidant protein) expression via enhancement of Nrf2 (NF-E2- related factor 2) activity which implies that MCL may work as a neuroprotective substance in neurodegenerative disorders related to neuroinflammation [33]. MCL has been ascribed to deter DSS (dextran sodium sulphate)-mediated rheumatic arthritis [53], inflammatory enteric ailment, and colitis-associated tumors [54]. Still, the role of MCL in sepsis and microbial infection is uncertain. More efforts are required to screen the accurate molecular targets of MCL.

## 3 Anti-cancer activities of MCL

Cancer is a complicated condition that arises due to the combination of metabolic, signaling, genetic, and epigenetic anomalies that severely disturb cell growth's normal controls and programmed cell death [55]. The etiological process of cancer cell proliferation is assisted by specific chemical reaction pathways or sequences [56,57]. Compounds obtained from natural sources could effectively kill cancerous cells and act as therapeutic agents [58]. Nature is a good source of antitumor drugs extracted from natural sources [59,60]. This might be due to some vital phytochemicals having the capability of inhibiting many pathways and preventing metastasis, in addition to their key roles in the inhibition of cancerous cells [60,61]. Drugs are obtained in trace amounts from natural products; therefore, some other natural products can serve as a precursor for the partial chemical synthesis of the target drug. Chemoprevention of cancer by natural biochemically active agents like minerals, vitamins, and phytochemicals has emerged as a realistic and auspicious realm to reduce the cancer burden. This method is getting attention steadily. These natural compounds are multitargeted, unlike monotargeted pharmaceutical medicines, which regulate cell proliferation and growth [62]. At present, >60% of commercially available anticancer drugs have a natural origin encompassing plants, marine organisms, and microorganisms [63]. Plant secondary metabolites like terpenes, alkaloids, and polyphenols have been acknowledged well for their cytotoxic potential against various cancers [[64], [65], [66]]. Out of 55,000 (approx.) terpenes isolated up till now, only a narrow fraction has cancer eradication potential. Sesquiterpene lactones (SLs) as a prime group of terpenes, have been screened cytotoxic against a diverse array of cancers [67]. Terpenoid sesquiterpene lactones belong to the Asteraceae family which are known to possess multiple biological activities such as anti-bacterial, antimalarial, anti-cancer, antiviral, antioxidant, antifungal, and anti-inflammatory [15,68,69]. The anticancer activity of MCL was reported by different groups. Selenoprotein thioredoxin reductase was identified as an important cellular target of MCL, and MCL may target the redox system in cancer cells. Junmin Zhang et al. reported that targeting thioredoxin reductase by MCL contributes to radiosensitizing and inducing apoptosis of HeLa cells [70]. Zhongren Xu et al., investigated that MCL elicits ROS-mediated ERS-driven immunogenic cell death in hepatocellular carcinoma [71]. Jianshuang Guo et al., showed A rational foundation for MCL-based combination strategy by targeting redox and metabolic circuits in cancer cells [72]. ACT001, a fumarate salt form of MCL, also known as dimethylaminomicheliolide, *i.e.,* DMAMCL converts to MCL consistently under physiological conditions. Li Q et al. investigated the role of ACT001 in glioblastoma cell lines, the study concludes that ACT001 directly binds to IKKβ and inhibits its phosphorylation also modulates the NF-κB/MnSOD/ROS axis by targeting IKKβ to inhibit glioblastoma cell growth [73]. Hou Y et al. studied the targeting of glioma stem-like cells with ACT001 through inhibition of AEBP1/PI3K/AKT signaling [74]. DMAMCL sensitized cancer cells to a single fraction of radiotherapy in vitro by inducing apoptosis and DNA double-strand breaks. The combination of DMAMCL-sensitized radiotherapy with anti-PD-L1 ICB significantly enhanced antitumor efficacy by increasing tumor-infiltrating CD4^+^ and CD8^+^ T cells and establishing immune memory [18].

### 3.1 Micheliolide and cell cycle arrest

Natural biologically active mitosis inhibitors or cell cycle controllers are reported to be potential candidates for cancer treatments [75,76]. As cancerous cells are characterized by prompt and unrestrained cell division [77], these naturally derived compounds are auspiciously capable of deterring the activity of cyclins and cyclin-dependent kinases, together with several protein factors and enzymes, regulating the cell cycle, helping cells to attain a controlled state [78].

MCL has been recognized in human lung cancer cells to arrest the cell cycle at S and G1/G0 phases. In humans, Mouse Tumor-Associated Fibroblasts (H460) cells MCL causes arrest at the S + G1/G0 checkpoint, downregulating the Protein coding gene (Notch4) and upregulating cleaved caspase3 expression [79].

An adduct of MCL, DMAMCL, has been reported to arrest the cell cycle in hepatocellular carcinoma (HCC) and rhabdomyosarcoma (RMS) cell lines at G2/M and SubG1 phase respectively. DMAMCL increased the number of HCC cells at the G2/M stage. Downregulation of cyclin B1 and cyclin D and tumor metastasis proteins MMP-9 and MMP-2 suggest that DMAMCL arrests the cell cycle at the G2/M stage, inhibiting the cell invasion and migration in HCC (LO2, Hepatoblastoma cell line (HepG2), Human hepatoma cell line (SMMC-7721), Human hepatoma cell line (Hep3B), Human hepatoma cell line (Huh7) cells [80].

### 3.2 Micheliolide and apoptosis

Apoptosis or programmed cell death ensues through specific pathways; mitochondrial or intrinsic pathway and death receptor or extrinsic pathway [81]. Pioneering caspases get prompted by these pathways and activate effector caspases, essential executioners of planned cell death [82]. Cumulative indications by scientific researchers suggest that nature-derived compounds can induce apoptosis while leading cancer cells to death by tempering different cellular factors in intrinsic or extrinsic apoptotic pathways [11,12].

**Fig. 3:**
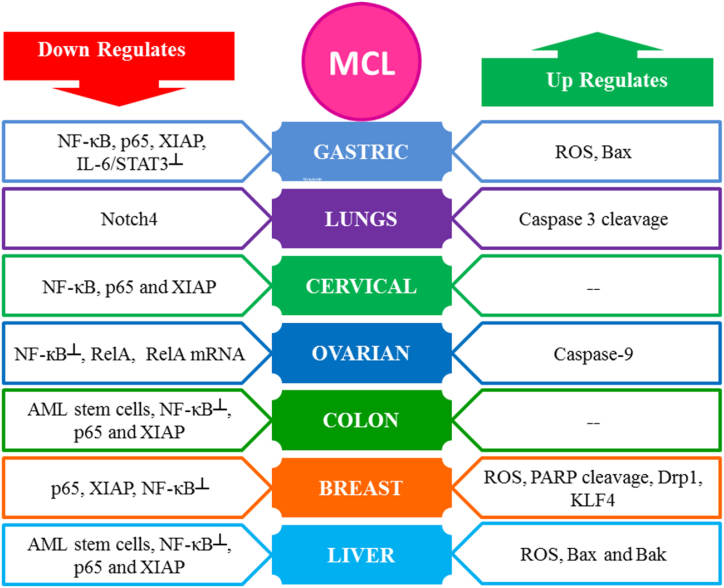
Diagrammatic illustration of anticancer activity of Micheliolide via interactions with multiple signaling pathways.

MCL has emerged as a novel paradigm due to its multi-targeted chemotherapeutic properties. Anti-cancer property of MCL has been stated to be associated with the inhibition of cell proliferation and apoptosis stimulation by oxidative stress/ROS accumulation [21,24,80,[83], [84], [85]], caspase family activation [21,24,79,80,83], down-regulation of anti-apoptotic proteins: Mcl-1 [86], and Bcl-2 [19,80], activation of pro-apoptotic proteins: Bax [19,21,30,80] and Bak [80], the downregulating expression level of Notch4 [79], inhibiting IL-6/STAT3 [86] and NF-κB pathway [20,23], downregulating PI3K/Akt/mTOR, JNK, Transcription factor (p65) and XIAP [20,21,80], inhibiting F-actin organization, enhancing PARP cleavage [83,84], activating PKM2 [85] and causing overexpression of Drp1 [84]. Table 2 shows the mechanism of action behind the cytotoxic activity of MCL, while the summary of the molecular targets is shown in Fig. 3.

**Table 2:** Cytotoxic effects attributed to Micheliolide in the case of certain cell lines and their molecular action mechanism.

Cancer	Cell Lines	Treatment Conditions		EC50/IC50	Molecular Targets	Cell Cycle Arrest	References
		No of Cells/Well	Time (hours)				
Lung	H460	____	48,72,96	30,60,90 (μmol/L)	Expression level of notch4 ↓, cleaved caspase3↑	G2/M, S phase and G1/G0	[79]
Liver	Hep G2, QGY-7703, Bel-7404, Hep3B, Huh7 and PLC/PRF/5	5 × 10^6^	24, 72	(30, 60) μM	ROS↑, F-actin organization┴, cleaved caspase-3↑, cleaved PARP↑	–	[83]
	Hep G2	10^4^	24, 48	9.61 (μg/ml)	NF-κB **┴**, p65 and XIAP↓	____	[20,35]
Colon	HCT 116	10^4^	24, 48	8.34 (μg/ml)	____	____	[35]
		1 × 10^5^ cells/mL	12, 48	12.68 μM	NF-κB**┴**, p65 and XIAP↓	____	[20]
Breast	MDA-MB-468	1 × 10^5^ cells/mL	12, 48	6.61 μM	NF-κB**┴**, p65 and XIAP↓	____	[20]
	MDA-MB-231			10.79 μM		____	
	MDA-MB-231, MCF-7	3 × 10^5^	24, 48	10 μM	Drp1↑, mitochondrial fission↑, ROS↑, PARP cleavage↑,	_____	[84]
	MCF-7	10^4^	24, 48	18.25 (μg/ml)	____	____	[35]
Ovarian	HeyA8,	___	72	(9.8 ± 2.2) μmol/L	NF-кB┴, caspase-9↑, RelA↓, RelA mRNA ↓,	____	[87]
	SKOV3			(12.0 ± 2.1) μmol/L			
	A2780/DDP			(12.8 ± 1.8) μmol/L			
Gastric	N87	5 × 10^4^	48	20 μM	IL-6/STAT3 ┴, IL-6↓, Bax ↑, XIAP↓, p-STAT3↓, cyclinD1↓, Mcl-1↓, MMP2↓, Ki67↓ and PCNA expression↓	_____	[86]
	AGS						
		1 × 10^5^ cells/mL	12, 48	11.75 μM	p65↓, XIAP↓, NF-κB pathway, and transcriptional activity┴	_____	[20]
Cervical	Hela	1 × 10^5^ cells/mL	12, 48	10.24 μM	p65↓, XIAP↓, NF-κB pathway, and transcriptional activity┴	_____	[20]
AML	HL-60	_____	_____	5.5 ± 1.4 μM	_____	_____	[18]
	HL-60/A			6.2 ± 2.2 μM			
Glioblastoma	U118MG	4 × 10^3^	24,48, 72	4.2 μM	ROS↑, Akt/mTOR┴, PKM2^Act^	____	[85]
	U251MG			14.5 μM			
	SF126			11.1 μM

### 3.3 Micheliolide and PI3K/Akt/NF-κB pathways

NF-κB is a family of transcription factors controlling the expression of several genes responsible for angiogenesis, survival, proliferation, immune responses, development, invasion, and metastasis. It is generally endorsed as the most common signaling regulator for the instigation and progress of cancer, supporting tumor cell growth through the expression of several growth regulator genes like Cyclin-dependent kinase 2 (CDK2), cyclin D1, D2, D3, and cyclin E. Therefore, modulation of the NF-κB pathway can be a novel paradigm for fighting cancer [88]. Phytochemicals can modulate the transcription factor NF-κB, hence, stimulating apoptosis [89]. A recent study showed that MCL can form a stable covalent bond with the cysteine residues of STAT3 and STAT5 to suppress their activation, thus inhibiting JAK/STAT signaling. MCL can weaken the NFκB pathway activity and inflammatory responses and increase cellular reactive oxygen species (ROS) to induce endoplasmic reticulum (ER) stress [90]. Zeng et al. synthesized derivatives of parthenolide and MCL and evaluated these compounds for in vivo anticancer activity in mice. The MCL derivative 9-oxomicheliolide showed promising anticancer activity. The mechanism studies revealed that 9-oxomicheliolide exhibited an inhibition effect against NF-κB and STAT3 signaling pathways, as well as induction effects of cell apoptosis [91]. Juan Yang et al., investigated the antitumor effect of the novel agent MCL/ACT001 in pancreatic ductal adenocarcinoma (PDAC), inducing cell apoptosis, cell migration, and ROS accumulation in vitro. Overall, their mechanistic investigations revealed that MCL exerted its antitumor activity via regulation of the EGFR-Akt-Bim signaling pathway, thus inducing Bim expression both in vitro and in vivo [93].

MCL inhibits NF-κB expression in leukemic cells. The two-fold elevated level of NF-κB was observed in primary acute myeloid leukemia (AML) mononuclear cells (MNCs) compared to normal MNCs collected from umbilical cord blood. Selectively induced NF-κB in leukemia stem cells (LSCs) was significantly intimidated after MCL treatment. Hence, NF-κB suppression is necessary for MCL-induced apoptotic death [23].

MCL downregulated the activity of NF-κB subunit p65, raised the *p*-PTEN level, and simultaneously mitigated the caspase-3, cardiac PI3K, phosphorylated Bad, and phosphorylated Akt intensities. Furthermore, the gene expressions of IL-1β (interleukin 1 beta), TNF-α (tumor necrosis factor-alpha), and tissue levels of MDA (malondialdehyde) were also downregulated after MCL treatment [94].

## 4 Other biological activities of MCL

Yaqin et al., presented their work that MCL competitively inhibited the binding of Interleukin-11 (IL-11) with Interleukin-11 Receptor-Alpha (IL-11Rα1), suppressing the activation of STAT3 and extracellular signal-regulated kinase 1/2-metatherian pathways, ultimately inhibiting renal tubular EMT and interstitial fibrosis induced by IL-11 [92]. Ziyang Gan et al., showed that MCL treatment may inhibit excessive osteoclast bone resorption without affecting bone formation in estrogen deficiency mice, and additionally, MCL could inhibit osteoclast formation via inhibiting P38 MAPK signaling pathway, and P79350 (a P38 agonist) could rescue this effect [95]. Shuting Li et al., reported that MCL impeded Brahma-related gene 1 (BRG1) from recognizing and attaching to histone H3 lysine 14 acetylation by binding to the asparagine (N1540) of BRG1, thus restraining fibrotic responses and TGF-β1-Smad2/3 signaling pathway. Their study showed that MCL targeting the N1540 residue of BRG1 may be a novel therapeutic strategy to combat PD-related peritoneal fibrosis [96]. Luo X. et al., reported that MCL attenuates atherosclerosis by suppressing macrophage ferroptosis via targeting KEAP1/NRF2 interaction [97]. Yang et al., reported that MCL attenuates neuroinflammation to improve cognitive impairment of Alzheimer's disease by inhibiting NF-κB and PI3K/Akt signaling pathways [98]. Luo et al. studied ACT001 which is a prodrug of MCL and may ameliorate ionizing radiation-induced lung injury by inhibiting the NLRP3 inflammasome pathway [99]. A study reported the MCL treatment significantly ameliorated radiation-induced intestinal tissue damage, inflammatory cell infiltration, and proinflammatory cytokine release. Furthermore, MCL-mediated induction of autophagy can ameliorate radiation-induced enteropathy [100]. Ackun-Farmmer et al. synthesized MCL analogs in bone-targeted polymeric nanoparticles (NPs) to improve bone marrow delivery. Following the treatment with MCL-based NPs therapies, a 13% improvement in median survival was seen with a 34-fold decrease in bone marrow leukemic stem cells as compared to controls [101]. A study published in 2020 by Alwaseem et al., provides insights into the biomolecular targets and mode of action of MCL in leukemia cells. This work highlights the usefulness of the current P450-mediated C–H functionalization approach for speeding up late-stage diversification and clarifying the biomolecular targets of MCL [102].

## 5 Conclusion

This review article aims to signify the pharmacological properties of this biologically active constituent from the plant family Magnoliaceae. This compound's most eminent medicinal properties are due to its anti-inflammatory and anticancer potential. Several scientific investigations suggest it modulates signaling cascades like PI3K/Akt and NF-κB. It is also an effective apoptosis inducer and arrests the cell cycle at G1/G0, S, and G2/M phases. The research area in cell cycle arrest also lacks evidence about cyclins and cyclin-dependent kinases. Moreover, exact action mechanisms should also be investigated by various techniques as well as experimental studies. The efficacy of Micheal's adducts of MCL and DMAML, suggests that its pharmacological potential may be enhanced by making its various derivatives and investigating their potential against many diseases especially cancer, the second deadliest malignancy worldwide. According to our suggestions, toxicological profiling of MCL is also needed to potentiate it as a novel anticancer and anti-inflammatory agent in clinical trials.

## Credit authorship contribution statement

**Jalal Uddin:** Supervision, Funding acquisition. **Mehwish Fatima:** Writing – original draft. **Ammara Riaz:** Supervision, Conceptualization. **Ghulam Mustafa Kamal:** Writing – review & editing. **Abdullatif Bin Muhsinah:** Funding acquisition. **Abdul Razzaq Ahmed:** Funding acquisition. **Ramsha Iftikhar:** Writing – review & editing.

## Declaration of competing interest

The authors declare that they have no known competing financial interests or personal relationships that could have appeared to influence the work reported in this paper.

## List of Abbreviations

PI3K/Akt pathway: Phosphoinositide-3-kinase–protein kinase B/Akt
NF-κB pathway: Nuclear factor kappa B
TLR4: Toll-like receptor 4
NSAIDs: Non-steroidal type anti-inflammatory drugs
TNF-α: Tumor necrosis factor alpha
MCP-1: Monocyte chemoattractant protein-1
IL-6: Interleukin 6
IL-10: Interleukin 10
IFN-β: Interferon β
RAW 264.7: Monocyte/macrophage cell line
CD14^+^: Cluster of differentiation 14
THP-1: Human leukemia monocytic cell line
AML12: Alpha mouse liver 12
PPAR-γ: Peroxisome proliferator-activated receptor gamma
*p*-IкBα: Anti-phosphorylated-IκBα
AMPK/mTOR: Adenosine monophosphate-activated protein kinase/Mammalian target of rapamycin
HO-1: Heme oxygenase-1
Nrf2: NF-E2- related factor 2
H460: Mouse Tumor-Associated Fibroblasts
HepG2: Hepatoblastoma cell line
SMMC-7721: Human hepatoma cell line
Hep3B: Human hepatoma cell line
Huh7: Human hepatoma cell line
Bax: Bcl-2-associated X protein
Bak: Proapoptotic effector Bcl-2 proteins
Notch4: Protein Coding gene
JNK: c-Jun *N*-terminal kinases
p65: Transcription factor
XIAP: X-linked inhibitor of apoptosis protein
PARP: Poly (ADP-ribose) polymerases
PKM2: Pyruvate kinase M2
PTEN: Phosphatase and tensin homologue
MAPK: Mitogen-activated protein kinase
STAT3: Signal transducer and activator of transcription 3
Wnt: Wingless and Int-1 (secreted factors that regulate cell growth, motility, and diﬀerentiation during embryonic development
TGFβ: Transforming growth factor-β
EGFR: Epidermal growth factor receptor
